# Quantifying degrees of necessity and of sufficiency in cause‐effect relationships with dichotomous and survival outcomes

**DOI:** 10.1002/sim.8331

**Published:** 2019-08-06

**Authors:** Andreas Gleiss, Michael Schemper

**Affiliations:** ^1^ Section for Clinical Biometrics, Center for Medical Statistics, Informatics, and Intelligent Systems Medical University of Vienna Vienna Austria

**Keywords:** attributable risk, Cox regression, explained variation, logistic regression, necessary condition, sufficient condition

## Abstract

We suggest measures to quantify the degrees of necessity and of sufficiency of prognostic factors for dichotomous and for survival outcomes. A cause, represented by certain values of prognostic factors, is considered necessary for an event if, without the cause, the event cannot develop. It is considered sufficient for an event if the event is unavoidable in the presence of the cause. Necessity and sufficiency can be seen as the two faces of causation, and this symmetry and equal relevance are reflected by the suggested measures. The measures provide an approximate, in some cases an exact, multiplicative decomposition of explained variation as defined by Schemper and Henderson for censored survival and for dichotomous outcomes.

The measures, ranging from zero to one, are simple, intuitive functions of unconditional and conditional probabilities of an event such as disease or death. These probabilities often will be derived from logistic or Cox regression models; the measures, however, do not require any particular model.

The measures of the degree of necessity implicitly generalize the established attributable fraction or risk for dichotomous prognostic factors and dichotomous outcomes to continuous prognostic factors and to survival outcomes. In a setting with multiple prognostic factors, they provide marginal and partial results akin to marginal and partial odds and hazard ratios from multiple logistic and Cox regression.

Properties of the measures are explored by an extensive simulation study. Their application is demonstrated by three typical real data examples.

## 1 INTRODUCTION

The investigation of possible causes for the development of diseases and for outcomes of diseased individuals is a main task of medical research. In clinical medicine, assumed cause‐effect relationships are typically explored by means of the statistical tool box for prognostic factor studies. Naturally, epidemiologists have also been interested in the field of causation for a long time and have introduced useful concepts.^1^ However, application of these concepts has been hampered by the limited availability of statistical measures (except for attributable fractions) and corresponding estimation of these measures for target populations (cf the work of Vanderweele^2^). The methodology presented here increases the versatility of the early concepts of necessary and of sufficient conditions to empirical research.

While the statistical literature offers numerous approaches to analyze different aspects of the effects of prognostic factors on dichotomous or survival outcomes, here we focus on an aspect that has received little attention: the degree to which levels of a prognostic factor are either necessary or sufficient for a (un)favorable outcome.

Let the level of interest of a dichotomous factor alternatively be termed *cause* or *condition*. Such a condition can be necessary and/or sufficient for a (un)favorable outcome. A condition is considered *necessary* for a (un)favorable outcome, eg, disease or complete cure from disease, if without the condition, the outcome cannot develop. A condition is considered *sufficient* for a (un)favorable outcome if this outcome is unavoidable in the presence of the condition. Necessity and sufficiency can be seen as the two faces of causation,^3^ and this symmetry and equal relevance are reflected by the suggested measures.

If a condition is both necessary and sufficient, it completely determines the outcome. In other words, if and only if this condition is present, the outcome will develop. While there are examples for the latter case in medicine (eg, Tangier disease^4^), causation in this strict sense rarely is observed in clinical or epidemiological studies. Therefore, it is of medical interest to quantify the degree to which the effect of a condition or, more generally, of a prognostic factor is necessary or sufficient for an outcome.

We think that measures for this purpose should have the following properties (cf the works of Kvålseth^5^ and Schemper and Stare^6^):

1. Simplicity and intuitively clear interpretation; a range from 0 to 1 being desirable, the endpoints marking minimal and maximal necessity and sufficiency, respectively.
2. The measures of necessity and of sufficiency should be of similar structure but independently sensitive to relevant properties of datasets.
3. The measures should satisfy simple relationships to a corresponding measure of explained variation, given their conceptual proximity to the latter.
4. Availability for any type of prognostic factor, ie, continuous, dichotomous, and polytomous.
5. Availability for any type of regression model or prediction tool.

The measures *DN* and *DS* for the degrees of necessity and of sufficiency, respectively, introduced in the next Section, obey these criteria.

We want to motivate the use of such measures by means of a simple example: Nilsson et al^7^ studied the relationship between smoking and death due to lung cancer after a 33‐year follow‐up in a cohort of 12 664 males in Sweden. See Table 1.

**Table 1:** Data of 12 664 males in Sweden^7^

	Never smoked	Ever smoked	Total
Died from lung cancer	36	177	213
Otherwise	8120	4331	12 451
Total	8156	4508	12 664

The data confirm that smoking is quite *necessary* for a lung cancer death (most deaths occurred among smokers); however, smoking is not *sufficient* for death due to lung cancer (most of the smokers did not die from lung cancer). It would be desirable to quantify the degrees of necessity, *DN*, and of sufficiency, *DS*, as additional aspects of the factor smoking, additional, that is, to the relative risk (8.895) or to explained variation^8^ (*EV* = 0.017). According to the formulae of Section 2, values of *DN* and *DS* are 0.738 and 0.023, respectively. Though smoking increases the risk of death due to lung cancer by almost 9‐fold, it explains only less than 2% of the variability in the outcome. In Section 2, we shall learn how *EV* is connected to *DN* and *DS*. In our example, it is the low *DS* that is responsible for the low *EV*. Seen as an aspect or attribute of any prognostic factor, *DN* and *DS* will also be defined for continuous factors.

Sections 2 and 3 provide definitions for *DN* and *DS* for dichotomous and survival outcomes, respectively. In Section 4, the performance of *DN* and *DS* under typical and also under extreme situations is explored by artificial and by simulated data examples. We illustrate application to typical prognostic factor studies in Section 5 and close the paper with a discussion in Section 6.

## 2 DEGREES OF NECESSITY AND OF SUFFICIENCY FOR A DICHOTOMOUS OUTCOME

For a dichotomous outcome, let *D* denote the event of interest (eg, disease) with probability P(*D*) ≠ 0, 1. For a prognostic factor *X*, we propose to define *DN* and *DS* in a population as

(1) $${DN}_{1}=\sqrt{E_{X<}{\left(\frac{P\left(D\right)-P\left(D\;|\;X\right)}{P\left(D\right)-0}\right)}^{2}}$$

and

(2) $${DS}_{1}=\sqrt{E_{X>}{\left(\frac{P\left(D\;|\;X\right)-P\left(D\right)}{1-P\left(D\right)}\right)}^{2}},$$

where E_*X*<_ and E_*X*>_ denote expectation conditional on {*X*: P(*D* | *X*) < P(*D*)} and on {*X*: P(*D* | *X*) > P(*D*)}, respectively. Note that the noninformative case of P(*D*| *X*) = P(*D*) contributes neither to *DN* nor to *DS*. Alternatively, we propose

(3) $${DN}_{2}=E_{X<}\left(\frac{P\left(D\right)-P\left(D\;|\;X\right)}{P\left(D\right)-0}\right)$$

and

(4) $${DS}_{2}=E_{X>}\left(\frac{P\left(D\;|\;X\right)-P\left(D\right)}{1-P\left(D\right)}\right).$$

In the following, we use *DN* and *DS* without subscript if referring to both variants of the measures.

### 2.1 Comments on definitions

For an unfavorable outcome event *D* (like disease), the range of values for *X* with P(*D* | *X*) < P(*D*) defines its *protective* levels since at these levels, the conditional disease probability falls below the unconditional one. For a favorable outcome, the definitions above still apply but the same range defines harmful levels of *X*. It is therefore important to clearly denote the level of the outcome to which *DN* and *DS* measures refer. In the remaining text, we assume an unfavorable outcome, if not explicitly stated otherwise, and designate levels of *X* with P(*D* | *X*) < P(*D*) as protective.

By explicitly using 0 in the denominators of (1) and (3), we emphasize that the actual departure of P(*D* | *X*) from P(*D*) is standardized to the maximal (hypothetical) departure when P(*D* | *X*) assumes a value of 0. Likewise, the actual departure of P(*D* | *X*) from P(*D*) is standardized by the denominators of (2) and (4), to the maximal (hypothetical) departure when P(*D* | *X*) assumes a value of 1. Thus, *DN* and *DS* achieve their maximal values of 1 if conditional disease probabilities are at the extreme values of 0 and 1, respectively. Repeating from Section 1, a *perfect necessary condition* for a disease requires that no diseased individuals are observed for protective levels of the risk factor. Likewise, a *perfect sufficient condition* for a disease requires that no healthy individuals are observed for harmful levels of the risk factor. If conditional and unconditional disease probabilities do not differ, then there are no informative observations in (1) to (4), resulting in values of 0, both for *DN* and *DS*.

By squaring the *kernels* (P(*D*) − P(*D* | *X*))/P(*D*) and (P(*D* | *X*) − P(*D*))/(1 − P(*D*)), within the conditional expectation, *DN*_1_ and *DS*_1_ give larger weight to extreme conditional probabilities (ie, close to 0 and 1, respectively), compared to *DN*_2_ and *DS*_2_. The square root in (1) and (2) permits interpretation on the probability scale with an intention similar to calculating standard deviations from variances. Since squaring is a convex function, Jensen's inequality gives *DN*_2_ ≤ *DN*_1_ and *DS*_2_ ≤ *DS*_1_. On the other hand, *DN*_2_ ≥ *DN*_1_^2^ and *DS*_2_ ≥ *DS*_1_^2^ since the kernels are constrained in [0, 1].

The definitions presented so far refer to marginal measures. As with *EV*, it is also possible and useful to define a *partial* degree of necessity as the difference between two *DN* measures for nested models (and analogously for *DS*). This makes it possible to quantify the gain in *DN* or *DS* when adding further prognostic factors to a model.

### 2.2 Connection to attributable risk

The reader will notice that the kernel of *DN* is the *attributable risk* for a single dichotomous prognostic factor *X*. The attributable risk, *AR* = (P(*D*) − P(*D* | *X* = 0))/P(*D*), quantifies the relative extent to which the disease probability can be reduced by shifting all subjects to the unexposed level (*X* = 0), ie, by eliminating the exposure.^9^ In this way, high values of *AR* indicate that the harmful level of a dichotomous risk factor tends to be necessary for the disease. For a general, not necessarily dichotomous, prognostic factor *X* with a protective value *x*, (P(*D*) − P(*D* | *X* = *x*))/P(*D*) gives the relative reduction in disease probability, if all subjects are hypothetically shifted to *x*. Thus, *DN* in (1) and (3) can be seen as averages of *AR*‐type measures across all protective levels of *X*. Alternative extensions of *AR* to nondichotomous prognostic factors have been proposed.^10^, ^11^

As already noted by Nelson and O'Brien,^4^ *AR* only covers the necessity part of causation. Therefore, for a single dichotomous prognostic factor *X*, we define the *reverse attributable risk*, *AR*^∗^ = (P(¬*D*) − P( ¬ *D* | *X* = 1))/P(¬*D*), as the relative reduction of the probability of “nondisease” (¬*D*) by shifting all subjects into the exposed level (*X* = 1). High values of AR* indicate that moving subjects to the exposed level will substantially increase the disease probability. For a general prognostic factor *X* with a harmful value *x*,

$$\frac{\left[1-P\left(D\right)\right]-\left[1-P\left(D\;|\;X=x\right)\right]}{\left[1-P\left(D\right)\right]}=\frac{P\left(D\;|\;X=x\right)-P\left(D\right)}{1-P\left(D\right)}$$

gives a suitably normalized increase in disease probability, if all subjects are hypothetically shifted to *x*. Thus, *DS* in (2) and (4) can be seen as averages of *AR* ^∗^‐type measures across all harmful levels of *X*.

### 2.3 Explained variation as constituted by the degrees of necessity and of sufficiency

In this section, we investigate the contribution of necessity and of sufficiency to explained variation. For dichotomous outcomes, the population definition of the *indirect measure of explained variation* 8 is

(5) $$EV=1-\frac{E_{X}\left[P\left(D\;|\;X\right)\left(1-P\left(D\;|\;X\right)\right)\right]}{P\left(D\right)\left(1-P\left(D\right)\right)}.$$

*EV* quantifies the relative reduction in predictive inaccuracy when unconditional prediction is replaced by conditional prediction.^8^ We assume that P(*D*) = E_*X*_(P(*D* | *X*)), which is fulfilled in practice if conditional probabilities are estimated by a calibrated model. Then, the numerator of (5) can be transformed to

$$\begin{array}{ll} & P\left(D\right)\left(1-P\left(D\right)\right)-E_{X}\left[P\left(D\;|\;X\right)\left(1-P\left(D\;|\;X\right)\right)\right]\\ & =E_{X}\left(P\left(D\;|\;X\right)\right)-{\left(P\left(D\right)\right)}^{2}-E_{X}\left(P\left(D\;|\;X\right)\right)+E_{X}{\left(P\left(D\;|\;X\right)\right)}^{2}\\ & =E_{X}{\left(P\left(D\;|\;X\right)\right)}^{2}-2P\left(D\right)E_{X}\left(P\left(D\;|\;X\right)\right)+{\left(P\left(D\right)\right)}^{2}\\ & =E_{X}{\left(P\left(D\;|\;X\right)-P\left(D\right)\right)}^{2} \end{array}$$

resulting in

(6) $$EV=\frac{E_{X}{\left(P\left(D\;|\;X\right)-P\left(D\right)\right)}^{2}}{P\left(D\right)\left(1-P\left(D\right)\right)}.$$

This reformulation shows that *EV* = 0 if and only if P(*D* | *X*) = P(*D*), which is also equivalent to *DN* = *DS* **=** 0. On the other hand, *DN* = 1 if and only if P(*D* | *X*) = 0 whenever P(*D* | *X*) < P(*D*), and *DS* = 1 if and only if P(*D* | *X*) = 1 whenever P(*D* | *X*) > P(*D*). Following from (5), these two conditions are together equivalent to *EV* = 1. Note that levels *x* with P(*D* | *X* = *x*) = P(*D*) are not only noninformative for *DN* and *DS* but also for *EV* using (6).

Let *α* = P_*X*_(P(*D* | *X*) > P(*D*)) denote the probability of a harmful level of *X*. By splitting the expectation E_*X*_(.) in (6) into conditional expectations E_*X<*_(.) and E_*X>*_(.), *EV* can be decomposed into simple functions of *DN*_1_ and *DS*_1_

(7) $$\begin{array}{cc} EV & =\left(1-\alpha \right)\frac{E_{X<}{\left(P\left(D\;|\;X\right)-P\left(D\right)\right)}^{2}}{P\left(D\right)\left(1-P\left(D\right)\right)}+\alpha \frac{E_{X>}{\left(P\left(D\;|\;X\right)-P\left(D\right)\right)}^{2}}{P\left(D\right)\left(1-P\left(D\right)\right)}\\ & =\left(1-\alpha \right)\frac{P\left(D\right)}{1-P\left(D\right)}{{DN}_{1}}^{2}+\alpha \frac{1-P\left(D\right)}{P\left(D\right)}{{DS}_{1}}^{2}. \end{array}$$

For P(*D*) = 0.5, this results in *EV* as a weighted average of the squared *DN*_1_ and *DS*_1_. In general, the weights (1‐α) and α are multiplied by the odds of disease and nondisease, respectively. Thus, the proportion of variation explained by *X* is closer to the squared degree of necessity induced by *X* if the odds for disease are high and there is a low probability of harmful levels for *X*. For example, in the lung cancer data presented in the introduction, the unconditional disease probability is estimated as 1.7%, which results in a much higher weight for *DS* in the decomposition of *EV* (despite an estimated value for α equal to 0.36). In general, Equation (7) shows that rare diseases (P(*D*) small) need highly sufficient prognostic factors to achieve a reasonably large value of *EV*.

For *DN*_2_ and *DS*_2_, no relation with *EV* similar to (7) can be deduced, since *EV* is based on squared deviations between conditional and unconditional disease probabilities, while *DN*_2_ and *DS*_2_ average over absolute differences. However, in Appendix A, we show that *EV* ≥ *DN*_2_ · *DS*_2_ while the empirical examples investigated in Sections 4 and 5 demonstrate that *DN*_1_ · *DS*_1_ usually is close to *EV*.

### 2.4 Estimation

For estimation of *DN* and *DS*, we replace population values in (1) to (4) by sample estimates. For a sample of *n* individuals, let ${\hat{p}}_{i}$ denote the estimate of the conditional disease probability P(*D* | *X*) for the *i*th individual. For example, the conditional prediction could be based on a (multiple) logistic regression model. Furthermore, let $\bar{p}$ denote an estimate of P(*D*) and *n*_<_ and *n*_>_ the number of observations *i* with ${\hat{p}}_{i}<\bar{p}$ and ${\hat{p}}_{i}>\bar{p}$, respectively. Then, *DN*_1_ and *DS*_1_ can be estimated by

(8) $${\hat{DN}}_{1}=\sqrt{\frac{1}{n_{<}}\sum_{{\hat{p}}_{i}<\bar{p}}{\left(\frac{\bar{p}-{\hat{p}}_{i}}{\bar{p}}\right)}^{2}}$$

and

(9) $${\hat{DS}}_{1}=\sqrt{\frac{1}{n_{>}}\sum_{{\hat{p}}_{i}>\bar{p}}{\left(\frac{{\hat{p}}_{i}-\bar{p}}{1-\bar{p}}\right)}^{2}}.$$

Similarly,

(10) $${\hat{DN}}_{2}=\frac{1}{n_{<}}\sum_{{\hat{p}}_{i}<\bar{p}}\left(\frac{\bar{p}-{\hat{p}}_{i}}{\bar{p}}\right)$$

and

(11) $${\hat{DS}}_{2}=\frac{1}{n_{>}}\sum_{{\hat{p}}_{i}>\bar{p}}\left(\frac{{\hat{p}}_{i}-\bar{p}}{1-\bar{p}}\right).$$

These point estimates can be accompanied by bootstrap confidence intervals. We recommend calculating percentile or BCa bootstrap confidence intervals.^12^ As usual, bootstrap replicates must be taken on the level of the full vector of observations, and the algorithm or model producing the ${\hat{p}}_{i,b}$ must be applied to each bootstrap replicate *b* = 1,…,*B*. Within each bootstrap replicate, the estimate of *DN* and *DS* must be set to zero whenever the mean of ${\hat{p}}_{i,b}$ over the harmful *X* range of the original sample falls below the mean over the protective range. In this way, we prevent switching the meaning of protective/harmful between the original sample and the bootstrap replicates.

### 2.5 Relationships for a single dichotomous prognostic factor

In the case of a single dichotomous prognostic factor, the data can be arranged as shown in Table 2, where *a*, *b*, *c*, and *d* denote the absolute frequencies in the respective cells with *a* + *b* + *c* + *d* = *n*.

**Table 2:** Notation for a 2 x 2 setting

Outcome			
*D*	*c*	*d*	
¬*D*	*a*	*b*	
	0	1	*X*

The disease probability is estimated as $\bar{p}=\left(c+d\right)/n$. If *ad* − *bc* > 0, then *c*/(*a*+*c*) < (*c*+*d*)/*n*, and therefore *X* = 0 is the protective level. Inserting ${\hat{p}}_{i}=c/\left(a+c\right)$ for ${\hat{p}}_{i}<\bar{p}$ and ${\hat{p}}_{i}=d/\left(b+d\right)$ for ${\hat{p}}_{i}>\bar{p}$ into (8) to (11), we therefore get

$${\hat{DN}}_{1}={\hat{DN}}_{2}=\frac{ad-bc}{\left(a+c\right)\left(c+d\right)}\;\text{and}\;\hat{{DS}_{1}}=\hat{{DS}_{2}}=\frac{ad-bc}{\left(a+b\right)\left(b+d\right)}.$$

Note that *c* = 0 and *b* = 0 lead to 100% degrees of necessity and of sufficiency, respectively. If *ad* − *bc* < 0, the protective level is *X* = 1 such that

$${\hat{DN}}_{1}={\hat{DN}}_{2}=\frac{bc-ad}{\left(b+d\right)\left(c+d\right)}\;\text{and}\;\hat{{DS}_{1}}=\hat{{DS}_{2}}=\frac{bc-ad}{\left(a+b\right)\left(a+c\right)}.$$

Similarly, for *ad* − *bc* > 0, we get

$$\hat{AR}=\frac{ad-bc}{\left(a+c\right)\left(c+d\right)}\;\text{and}\;{\hat{AR}}^{*}=\frac{ad-bc}{\left(a+b\right)\left(b+d\right)}.$$

Inserting into (5) or (6) results in

$$\hat{EV}=\frac{{\left(ad-bc\right)}^{2}}{\left(a+b\right)\left(a+c\right)\left(b+d\right)\left(c+d\right)},$$

which is identical with the multiple *R* ^2^ for dichotomous outcomes^13^, ^14^ and with explained variation measures ${\tilde{V}}_{B}$ and ${\hat{V}}_{B}$ (see Section 2.2 in the work of Schemper^8^). With analogous derivations on the population level, we therefore have *DN*_1_ = *DN*_2_ = *AR*, *DS*_1_ = *DS*_2_ = *AR^∗^*, and *EV* = *DN* · *DS* = *AR* · *AR*^∗^. Thus, in the 2 x 2 case, explained variation can exactly be decomposed as the product of the degrees of necessity and of sufficiency.

The estimates $\bar{p}$ and ${\hat{p}}_{i}$ above rely on data from representative samples, often available from cross‐sectional and cohort studies, usually not from case‐control studies.

## 3 DEGREES OF NECESSITY AND OF SUFFICIENCY FOR A SURVIVAL OUTCOME

While, for dichotomous outcomes, we compare single conditional and unconditional probabilities of an event for each individual, with survival outcomes conditional and unconditional cumulative distribution functions, *F*(*t*) and *F*(*t*| *X*), are compared.

For a single time *t*, the situation is identical to dichotomous outcomes dealt with in Section 2, and the same measures of necessity and sufficiency can be used. However, we are usually interested in survival over the full follow‐up range [0, *τ*], and hence, it is natural to average over *t* (0 ≤ *t* ≤ *τ*)

(12) $$\;{DN}_{1}\left(\tau \right)={\left\{\int_{0}^{\tau }f\left(t\right)\mathrm{dt}\right\}}^{-1}\int_{0}^{\tau }{\left[E_{X<}{\left(\frac{F\left(t\right)-F\left(t|X\right)}{F\left(t\right)}\right)}^{2}\right]}^{1/2}f\left(t\right)\mathrm{dt}$$

(13) $${DS}_{1}\left(\tau \right)={\left\{\int_{0}^{\tau }f\left(t\right)\mathrm{dt}\right\}}^{-1}\int_{0}^{\tau }{\left[E_{X>}{\left(\frac{F\left(t\;|\;X\right)-F\left(t\right)}{1-F\left(t\right)}\right)}^{2}\right]}^{1/2}f\left(t\right)\mathrm{dt},$$

where *f*(*t*) is the density of events in time. The square root of the terms in brackets defines *DN*_1,*t*_ and *DS*_1,*t*_, the degrees of necessity and of sufficiency at time *t*, respectively. They are analogous to definitions (1) and (2) of *DN*_1_ and *DS*_1_ with dichotomous outcomes. Furthermore, E_*X*<_ and E_*X>*_ denote expectation conditional on {*X*: *F*(*t* | *X*) < *F*(*t*)} and on {*X*: *F*(*t* | *X*) > *F*(*t*)}, respectively.

For a single dichotomous prognostic factor, the kernel (*F*(*t*) − *F*(*t* | *X* = 0))/*F*(*t*) was termed *attributable risk function* to quantify the proportion of disease that would be prevented at time *t* by a fully effective intervention.^15^, ^16^ A generalization to “with‐intervention” distributions was proposed by Samuelsen and Eide.^17^

In definitions (12) and (13), *DN*_1,*t*_ and *DS*_1,*t*_ are appropriately weighted by *f*(*t*) within the time range of follow‐up or of medical interest. The same approach to averaging an effect‐size measure over time has been used by Schemper and Henderson,^18^ which also permits comparability of *DN*_1_(*τ*) and *DS*_1_(*τ*) with their explained variation measures for survival.

Similarly, we define

$${DN}_{2}\left(\tau \right)={\left\{\int_{0}^{\tau }f\left(t\right)\mathrm{dt}\right\}}^{-1}\int_{0}^{\tau }\left[E_{X<}\left(\frac{F\left(t\right)-F\left(t|X\right)}{F\left(t\right)}\right)\right]f\left(t\right)\mathrm{dt}$$

and

$${DS}_{2}\left(\tau \right)={\left\{\int_{0}^{\tau }f\left(t\right)\mathrm{dt}\right\}}^{-1}\int_{0}^{\tau }\left[E_{X>}\left(\frac{F\left(t\;|\;X\right)-F\left(t\right)}{1-F\left(t\right)}\right)\right]f\left(t\right)\mathrm{dt}.$$

As with dichotomous outcomes, the two variants coincide for dichotomous *X*, ie, *DN*_1_(*τ*) = *DN*_2_(*τ*) and *DS*_1_(*τ*) = *DS*_2_(*τ*).

In a given sample, let *t*_*i*_, *η*_*i*_, and *x*_*i*_ denote observation time, censoring indicator, and vector of prognostic factors, respectively, for individual *i* (1 ≤ *i* ≤ *n*). Assume there are *m* distinct survival times in the sample, at times *t*_(*j*)_ (1 ≤ *j* ≤ *m*), with *d*_*j*_ deaths at *t*_(*j*)_. Then, at each distinct death time *t*_(*j*)_, we estimate the degree of necessity as

$${\hat{DN}}_{1,t_{\left(j\right)}}={\left[\frac{1}{n_{<}\left(t_{\left(j\right)}\right)}\sum_{i=1}^{n}I\left(\hat{F}\left(t_{\left(j\right)}\;|\;x_{i}\right)<\hat{F}\left(t_{\left(j\right)}\right)\right){\left(\frac{\hat{F}\left(t_{\left(j\right)}\right)-\hat{F}\left(t_{\left(j\right)}\;|\;x_{i}\right)}{\hat{F}\left(t_{\left(j\right)}\right)}\right)}^{2}\right]}^{1/2}$$

with *I*(.) denoting the indicator function and *n*_<_(*t*_( *j*)_) the number of subjects *i* with $\hat{F}\left(t_{\left(j\right)}\;|\;x_{i}\right)<\hat{F}\left(t_{\left(j\right)}\right)$ at time *t*_( *j*)_.

In practice, the required unconditional and conditional estimates, $\hat{F}\left(t_{\left(j\right)}\right)=1-\hat{S}\left(t_{\left(j\right)}\right)$and $\hat{F}\left(t_{\left(j\right)}\;|\;x_{i}\right)=1-\hat{S}\left(t_{\left(j\right)}\;|\;x_{i}\right)$, are most often obtained by means of the Kaplan‐Meier estimator^19^ for the unconditional and Cox regression^20^ for the conditional survival function estimate, $\hat{S}\left(t\right)$ and $\hat{S}\left(t\;|\;x_{i}\right)$, respectively. However, they might as well be obtained from other estimators and regression models such as parametric survival models.

To obtain an overall estimate of *DN*_1_, we form weighted averages of the ${\hat{DN}}_{1,t_{\left(j\right)}}$ over survival times, with weights designed to compensate the attenuation in observed death due to earlier censorship

$$\hat{{DN}_{1}}=w^{-1}\sum_{j}\hat{G}{\left(t_{\left(j\right)}\right)}^{-1}d_{j}{\hat{DN}}_{1,t_{\left(j\right)}}$$

with $w=\sum_{j}\hat{G}{\left(t_{\left(j\right)}\right)}^{-1}d_{j}$, and $\hat{G}\left(t\right)$ denoting the Kaplan‐Meier estimator of the censoring or “potential follow‐up” distribution estimated like *S*(*t*) but with the meaning of the censoring indicator *η* reversed.^21^ This type of weighting has also been used with explained variation measures for survival outcomes^18^ and permits consistent estimates of *DN*_1_(*τ*), as given by (12), in the presence of clinical or administrative censoring before *τ* (as will be confirmed by simulation results of Section 4.2).

Analogously to $\hat{{DN}_{1}}$, we estimate

$$\begin{array}{ll} {\hat{DS}}_{1} & =w^{-1}\sum_{j}\hat{G}{\left(t_{\left(j\right)}\right)}^{-1}d_{j}{\left[\frac{1}{n_{>}\left(t_{\left(j\right)}\right)}\sum_{i=1}^{n}I\left(\hat{F}\left(t_{\left(j\right)}\;|\;x_{i}\right)>\hat{F}\left(t_{\left(j\right)}\right)\right){\left(\frac{\hat{F}\left(t_{\left(j\right)}\;|\;x_{i}\right)-\hat{F}\left(t_{\left(j\right)}\right)}{1-\hat{F}\left(t_{\left(j\right)}\right)}\right)}^{2}\right]}^{1/2}\\ \hat{{DN}_{2}} & =w^{-1}\sum_{j}\hat{G}{\left(t_{\left(j\right)}\right)}^{-1}d_{j}\left[\frac{1}{n_{<}\left(t_{\left(j\right)}\right)}\sum_{i=1}^{n}I\left(\hat{F}\left(t_{\left(j\right)}\;|\;x_{i}\right)<\hat{F}\left(t_{\left(j\right)}\right)\right)\frac{\hat{F}\left(t_{\left(j\right)}\right)-\hat{F}\left(t_{\left(j\right)}\;|\;x_{i}\right)}{\hat{F}\left(t_{\left(j\right)}\right)}\right] \end{array}$$

and

$${\hat{DS}}_{2}=w^{-1}\sum_{j}\hat{G}{\left(t_{\left(j\right)}\right)}^{-1}d_{j}\left[\frac{1}{n_{>}\left(t_{\left(j\right)}\right)}\sum_{i=1}^{n}I\left(\hat{F}\left(t_{\left(j\right)}\;|\;x_{i}\right)>\hat{F}\left(t_{\left(j\right)}\right)\right)\frac{\hat{F}\left(t_{\left(j\right)}\;|\;x_{i}\right)-\hat{F}\left(t_{\left(j\right)}\right)}{1-\hat{F}\left(t_{\left(j\right)}\right)}\right].$$

Note that *DN* and *DS* are not affected by monotone transformation of the time scale, a property shared with the commonly employed semiparametric tools of survival analysis.

In the above definitions, the protective range of *X* is the set of values where the conditional distribution function estimate, ie, the conditional estimate of the cumulative incidence, is below the unconditional one. This is useful in the frequent case of survival outcomes defined as the time to an unfavorable event. In cases of a time to a favorable event, such as complete remission, the definitions above still apply but the protective (harmful) range of X would then comprise those values where the cumulative incidence is higher (lower) if estimates are conditional instead of unconditional. As with dichotomous outcomes, it is therefore important to clearly state the outcome to which degrees of necessity and of sufficiency refer.

## 4 EMPIRICAL RESULTS

In Sections 4.1 and 4.2, we investigate the ranges of *DN* and *DS* and their relationships with odds and hazard ratios and disease probability on the population level. In Section 4.3, we explore the amount of bias of the estimates of *DN* and *DS* for sample sizes likely encountered in practice.

### 4.1 Dichotomous outcome

We demonstrate the dependence of *DN*, *DS*, and *EV* on various constellations of a dichotomous outcome and a dichotomous prognostic factor on the population level by means of Table 3. The constellations can either be characterized by the probabilities of the cells, *a* to *d*, in a 2 x 2 table (see Table 2) or, equivalently, by the corresponding values of disease probability, P(*D*), the probability of the harmful level of *X*, *α*, and the odds ratio for disease, *OR* = (*ad*)/(*bc*). Depending on whether the *OR* is greater or smaller than 1, *α* = P(*X* = 1) or *α* = P(*X* = 0), respectively. Moreover, recall that both variants of *DN* and *DS*, *DN*_1_ and *DN*_2_, and *DS*_1_ and *DS*_2_ are identical in case of a dichotomous prognostic factor, and thus, subscripts for *DN* and *DS* are omitted.

**Table 3:** Population values of DN, DS, and EV for dichotomous outcome and dichotomous prognostic factor

**Scenario**	**P(D)**	**α**	**OR**	**a**	**b**	**c**	**d**	**DN**	**DS**	**EV**
1	0.1	0.1	10	84.1	5.9	5.9	4.1	0.346	0.346	0.120
2	0.1	0.5	10	48.9	41.1	1.1	8.9	0.787	0.087	0.069
3	0.1	0.9	10	9.9	80.1	0.1	9.9	0.878	0.011	0.010
4	0.5	0.1	10	48.9	1.1	41.1	8.9	0.087	0.787	0.069
5	0.5	0.5	10	38.0	12.0	12.0	38.0	0.519	0.519	0.270
6	0.5	0.9	10	8.9	41.1	1.1	48.9	0.787	0.087	0.069
7	0.9	0.1	10	9.9	0.1	80.1	9.9	0.011	0.878	0.010
8	0.9	0.5	10	8.9	1.1	41.1	48.9	0.087	0.787	0.069
9	0.9	0.9	10	4.1	5.9	5.9	84.1	0.346	0.346	0.120
10	0.1	0.1	100	87.4	2.6	2.6	7.4	0.716	0.716	0.513
11	0.1	0.5	100	49.9	40.1	0.1	9.9	0.975	0.108	0.106
12	0.1	0.9	100	10.0	80.0	0.0	10.0	0.988	0.012	0.012
13	0.5	0.1	100	49.9	0.1	40.1	9.9	0.108	0.975	0.106
14	0.5	0.5	100	45.5	4.5	4.5	45.5	0.818	0.818	0.669
15	0.5	0.9	100	9.9	40.1	0.1	49.9	0.975	0.108	0.106
16	0.9	0.1	100	10.0	0.0	80.0	10.0	0.012	0.988	0.012
17	0.9	0.5	100	9.9	0.1	40.1	49.9	0.108	0.975	0.106
18	0.9	0.9	100	7.4	2.6	2.6	87.4	0.716	0.716	0.513
19	0.1	0.1	0.1	5.9	84.1	4.1	5.9	0.346	0.346	0.120
20	0.1	0.5	0.1	41.1	48.9	8.9	1.1	0.787	0.087	0.069
21	0.1	0.9	0.1	80.1	9.9	9.9	0.1	0.878	0.011	0.010
22	0.5	0.1	0.1	1.1	48.9	8.9	41.1	0.087	0.787	0.069
23	0.5	0.5	0.1	12.0	38.0	38.0	12.0	0.519	0.519	0.270
24	0.5	0.9	0.1	41.1	8.9	48.9	1.1	0.787	0.087	0.069
25	0.9	0.1	0.1	0.1	9.9	9.9	80.1	0.011	0.878	0.010
26	0.9	0.5	0.1	1.1	8.9	48.9	41.1	0.087	0.787	0.069
27	0.9	0.9	0.1	5.9	4.1	84.1	5.9	0.346	0.346	0.120
28	0.1	0.1	1	81.0	9.0	9.0	1.0	0.000	0.000	0.000
29	0.5	0.5	1	25.0	25.0	25.0	25.0	0.000	0.000	0.000
30	0.5	0.5	0	0.0	50.0	50.0	0.0	1.000	1.000	1.000
31	0.1	0.5	∞	50.0	40.0	0.0	10.0	1.000	0.111	0.111
32	0.5	0.5	∞	50.0	0.0	0.0	50.0	1.000	1.000	1.000
33	0.9	0.5	∞	10.0	0.0	40.0	50.0	0.111	1.000	0.111
34	0.01	0.5	100	50.0	49.0	0.01	1.0	0.980	0.010	0.010
35	0.5	0.01	100	50.0	0.01	49.0	1.0	0.010	0.980	0.010

Abbreviations: P(*D*), unconditional disease probability; *OR*, odds ratio; α, probability of harmful level of *X*; *a, b, c, d*, as by Table 2 but with probabilities (%).

We have included scenarios with *OR* = 100, which are rarely observed in practice. However, Pepe et al have nicely shown that odds ratios even larger than 100 are required to reliably discriminate between individuals who will experience an event and those who will not.^22^ By exploring the effect of extreme odds ratios (and hazard ratios in the following Section 4.2), we demonstrate that *DN* and *DS* values close to 1 are indeed achievable.

Population values of *DN* and *DS* in Table 3 are obtained by inserting population probabilities instead of sample frequencies into the definitions given in Section 2.5. From Table 3, we confirm and learn that

(i) *DN*, *DS*, and *EV* are in the range from 0 to 1.
(ii) *DN* · *DS* = *EV*; because of (i), this implies *EV* ≤  *min* (*DN*, *DS*).
(iii) *DN* = *DS* if *P*(*D*) = *α* for any value of *OR*.
(iv) If *OR* is replaced by *OR*^−1^, then *DN*, *DS*, and *EV* are unchanged; in this case, however, by definition of α, *a*, and *b* are exchanged as well as *c* and *d* (compare Scenarios 1‐9 with 19‐27). This is sensible because otherwise *DN* (or analogously *AR*) would be obtained for an assumed “harmful” level of *X* leading to fewer unfavorable outcomes than the “protective” level.
(v) If *OR* = 1, then *DN*, *DS*, and *EV* are 0 (Scenarios 28‐29).
(vi) If and only if *c* = 0, then *DN* = 1; if and only if *b* = 0, then *DS* = 1; if and only if *b* = 0 ∧  *c* = 0, then *EV* = 1 (given *OR* > 1; for *OR* < 1, *b* and *c* are replaced by *a* and *d* in the previous conditions; Scenarios 30‐33).
(vii) For a rare unfavorable outcome (*P*(*D*) = 0.01, Scenario 34), the harmful level of *X* is highly necessary for this outcome (*DN* = 98%); however, it is by no means sufficient (*DS* = 1%), ie, the harmful level of *X* does not at all imply a high probability of the unfavorable level of the outcome (despite an *OR* = 100; see also the lung cancer example in the Introduction). For a rare harmful exposure level (*α* = 0.01, Scenario 35), the harmful level of *X* is extremely sufficient for this outcome (*DS* = 98%); however, it is by no means necessary (*DN* = 1%), ie, the unfavorable outcome can easily occur also with the protective level of *X* (despite an *OR* = 100). In both cases, the resulting low value of *EV* indicates the existence of further important factors responsible for disease, which are more sufficient or necessary, respectively.

**Figure 1:**
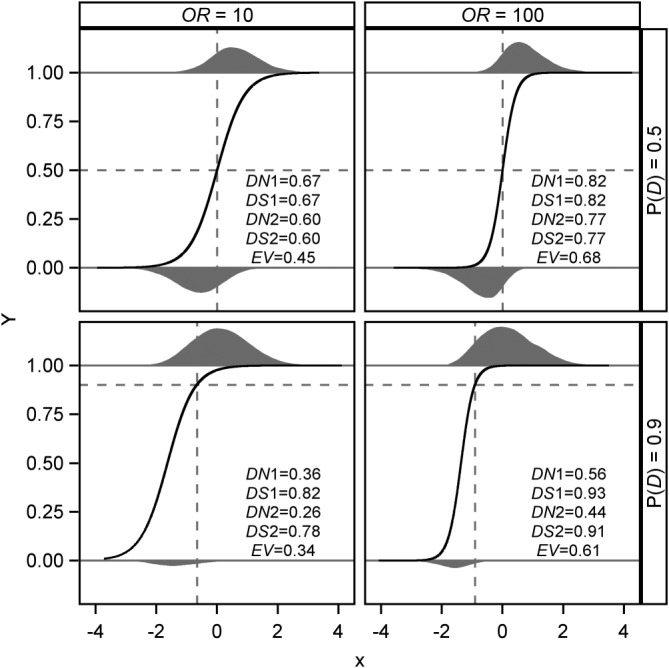
Population values of DN, DS, and EV for four scenarios with OR = 10, 100, P(D) = 0.5, 0.9, and standard normally distributed prognostic factor X. Below and above each of the corresponding panels, the distributions of X for Y = 0 and Y = 1 are shown, the areas of the smoothed histograms being proportional to 1 ‐ P(D) and P(D), respectively. Black solid line: logistic regression curve P(D | X); dashed horizontal line at P(D); dashed vertical line at X where P(D | X) = P(D), resulting in α = 0.50, 0.50, 0.75, 0.82 for upper left to lower right panel

The case of a dichotomous outcome and a continuous prognostic factor is illustrated by Figure 1, which demonstrates the effect of *P*(*D*) and *OR* on *DN*_1_, *DN*_2_, *DS*_1_, *DS*_2_, and *EV*. The results are based on a marginal standard normal distribution of *X*. Regression coefficients were set to *β*_1_ = log (10) and log (100), and *β*_0_ selected such that *P*(*D*) = 0.5 and 0.9, respectively. The conditional expectations in Equations (1) to (4) were numerically approximated using the *integrate*() function in R version 3.5.1.

The resulting population distributions of *X*, separately for *Y* = 0 and *Y* = 1, and logistic regression curves for P(*D* | *X*) are shown in Figure 1. For example, in the lower right subfigure for *OR* = 100 and *P*(*D*) = 0.9, P(*D* | *X*) is very close to 1 throughout the harmful range of *X* (right of the vertical dashed line), resulting in high contributions to *DS* in the kernel of definition (2). In contrast, *X* values in the protective range (left of the vertical dashed line) exhibit a large range for P(*D* | *X*), which leads to a medium value of *DN*.

As observed for a dichotomous prognostic factor, all three measures increase if *OR* increases from 10 to 100, and *DS*_1_ (*DS*_2_) and *DN*_1_ (*DN*_2_) increase and decrease, respectively, if increasing *P*(*D*). For *P*(*D*) = 0.5, the product of *DN*_1_ and *DS*_1_ exactly equals *EV* (upper panels in Figure 1). For *P*(*D*) ≠ 0.5, the departure of *DN*_1_ · *DS*_1_ from *EV* is smaller with *OR* = 100 (right panels in Figure 1) than with *OR* = 10. Further simulations show that, for extremely high (or low) *OR*, this discrepancy goes to zero, since an *OR* approaching infinity (or zero) produces a situation close to the 2 x 2 case (results not shown).

### 4.2 Survival outcome

For survival outcomes, we consider a dichotomous prognostic factor *X* and exponentially distributed survival times with hazards of exp(−*Xβ*) and *β* set to 0, −log(2), −log(10), and −log(100). This results in relative hazards of *HR* =  exp (−*β*) = 1, 2, 10, and 100. The Equations (12) and (13) on the population level were numerically approximated using the *integrate*() function in R version 3.5.1.

We considered two types of (noninformative) censoring: type I censoring at *τ* as a result of constant follow‐up of *τ* time units for all individuals; administrative censoring between 0 and *τ*, which results from uniformly distributed follow‐up when individuals enter a clinical trial at a constant rate over an interval from 0 to *τ*. In either case, data are analyzed at time *τ*. Results for *DN*, *DS*, and *EV*, the Schemper‐Henderson measure of explained variation,^18^ are presented in Table 4 with values of *τ* selected to give 0, 50, and 90% censoring under administrative censoring. Corresponding censoring percentages under type I censoring are lower. The purpose of producing results under both types of censoring for *DN* and *DS* is to distinguish the effect of changing *τ* from that of random censoring between 0 and *τ*. From Table 4, we learn that the expectations of *DN* and *DS* are virtually unaffected by additional (administrative) random censoring before *τ*. However, the measures are affected by the choice of *τ*, the maximum follow‐up time or maximum time of medical interest. Both properties are shared with the Schemper‐Henderson measure of explained variation for survival outcomes.^18^

**Table 4:** The DN, DS, and EV for survival outcome and balanced dichotomous prognostic factor

		Type 1 censoring				Administrative censoring			
HR	***τ***	% cens.	DN	DS	EV	% cens.	$\hat{\mathrm{DN}}$	$\hat{\mathrm{DS}}$	$\hat{\mathrm{EV}}$
1	‐	0	0.00	0.00	0.00				
	1.64	20	0.00	0.00	0.00	50	0.00	0.00	0.00
	0.22	80	0.00	0.00	0.00	90	0.00	0.00	0.00
2	‐	0	0.22	0.31	0.05				
	2.26	23	0.25	0.20	0.05	50	0.25	0.20	0.05
	0.30	80	0.32	0.04	0.02	90	0.33	0.04	0.02
10	‐	0	0.55	0.62	0.32				
	4.90	31	0.71	0.44	0.34	50	0.71	0.44	0.34
	0.43	81	0.80	0.09	0.09	90	0.80	0.09	0.10
100	‐	0	0.67	0.69	0.48				
	15.30	43	0.96	0.45	0.53	50	0.96	0.45	0.53
	0.47	81	0.98	0.11	0.14	90	0.98	0.10	0.13

Abbreviations: HR, hazard ratio; τ, time of maximum follow‐up; % cens., percentage of censored observations. For Type 1 censoring, the entries are population values, while under administrative censoring, they are based on simulated samples of 100 000 observations. Note that both variants of *DN* and of *DS* are identical for dichotomous prognostic factors.

As *DN* and *DS* can be considered as functions that vary with time, their dependence on *τ* is natural. Kejžar et al pointed out that explained variation measures for survival data that are unaffected by *τ* do this by implicitly extrapolating Cox regression coefficients beyond the time covered by the sample.^23^ The same would be the case with *DN*‐type and *DS*‐type measures that were unaffected by *τ*. However, Kejžar et al also demonstrate how the Schemper‐Henderson measure could be made independent of a particular τ, if extrapolation was justified (see also the work of Schemper and Kaider^24^). The same procedure can be used to make *DN* and *DS* unaffected by *τ*.

Table 5 shows that the distribution of *X* affects values of *DN* and *DS* in a similar way as presented for dichotomous outcomes in Table 3.

**Table 5:** Population values of DN, DS, and EV for uncensored survival outcome and dichotomous prognostic factor

	***α*** = 0.1			***α*** = 0.5			***α*** = 0.9		
HR	DN	DS	EV	DN	DS	EV	DN	DS	EV
1	0.00	0.00	0.00	0.00	0.00	0.00	0.00	0.00	0.00
2	0.05	0.47	0.02	0.22	0.31	0.05	0.36	0.08	0.02
10	0.16	0.85	0.12	0.55	0.61	0.32	0.80	0.21	0.12
100	0.24	0.94	0.17	0.68	0.68	0.48	0.93	0.25	0.17

Abbreviations: HR, hazard ratio; α, probability of harmful level of *X*.

### 4.3 Small sample results

While the main empirical properties of *DN* and *DS* can best be understood on the population level as dealt within the previous sections, in practice, only estimates of these measures are available. By means of simulation studies, we have explored bias and variability of estimates of *DN* and *DS* with sample sizes of *n* = 200 and *n* = 500 for several scenarios with dichotomous and survival outcomes. For each scenario investigated in this section, 250 random samples have been drawn using the specifications of Sections 4.1 and 4.2 for data generation.

For selected scenarios with dichotomous outcomes and dichotomous prognostic factor in Table 3, the distribution of bias in terms of its median and quartiles has been studied. Bias, calculated as sample minus population value, becomes appreciable if *P*(*D*) and *α* are unbalanced in opposite directions, eg, *P*(*D*) = 0.1 and *α* = 0.9, and the corresponding population value of *DN* or of *DS* is high. In addition, with population values of 0 for *DN* or for *DS*, appreciable bias (>0.1) is possible as estimates cannot become negative, a property shared by other bounded measures. Otherwise, no bias is observed. Detailed results are available in Table 1 of the Supplementary Material. The performance with continuous prognostic factors appears analogous as indicated in Table 2 of the Supplementary Material.

In Section 2.4, we have introduced confidence intervals for *DN* and *DS*, which might be useful given possible bias of point estimates. These provide the range of population values of *DN* and *DS* compatible with a given sample. We have explored the coverage of the recommended percentile and BCa intervals in a simulation study with 1000 simulated samples per scenario using 1000 bootstrap replicates per simulated sample. Coverage appears sufficiently close to the nominal level: eg, for underlying *DN* = 0, *P*(*D*) = 0.5, a standard normally distributed prognostic factor and *n* = 200, the two‐sided 95% confidence intervals covered the value 0 with a frequency of 94.8%. The average width of these confidence intervals is 0.19 for *n* = 200, and decreases to 0.12 for *n* = 500, to 0.09 for *n* = 1000, and to 0.03 for *n* = 10 000. For underlying *DN* = 0.5, the average width of these confidence intervals is 0.23, 0.14, 0.10, and 0.03, respectively. Simple percentile bootstrap intervals yielded very similar results. Though the use of *DN* and *DS* often will be descriptive only, these results can provide rough guidance on sample sizes required for medical studies reporting values of *DN* and *DS*.

The simulation studies on bias and variability of *DN* and *DS* for censored and uncensored survival outcomes (see Tables 3 and 4 of the Supplementary Material) indicate only negligible bias for underlying hazard ratios *HR* ≥ 2. The bias under *HR* = 1, where population *DN* and *DS* are 0, appears relatively small without censoring (≤0.04 for *n* = 200; decreasing for increasing sample size) but can increase substantially for high amount of censoring (eg, under 90% administrative censoring, ie, for expected 20 uncensored observations, it rises to estimated 0.15).

## 5 EXAMPLES

We demonstrate the application of our proposed measures to two datasets from prognostic factor studies, the first with a dichotomous, the second with a survival outcome. In Section 1, we already have presented a simple epidemiologic example with a single prognostic factor.

Datasets with multiple prognostic factors permit more elaborate usage of the proposed measures. The following two examples should give an impression of the usefulness of quantifying the degrees of necessity and of sufficiency, in addition to explained variation, and, of course, to standard results from logistic and Cox regression analysis. Both examples have already served to illustrate use of explained variation by models with dichotomous and survival outcomes.^8^

### 5.1 Prognostic factors for tumor penetration of prostatic capsule

A prostate cancer dataset from The Ohio State University Comprehensive Cancer Center has been presented in the work of Hosmer and Lemeshow.^25^ Statistical analysis of the 376 completely documented patients should determine whether potential prognostic factors measured at a baseline exam could be used to predict whether the tumor has penetrated the prostatic capsule. Penetration was observed in 41% of the patients. The following prognostic factors are considered: age (in years), race (White, Black), results of digital rectal exam (1 = no, 2 = unilobular, and 3 = bilobular nodule), detection of capsular involvement (no, yes), prostatic specific antigen value (PSA, in mg/ml, range 0.3‐139.7), tumor volume from ultrasound (in cm^3^, range 0‐97.6), and total Gleason score (range 0‐9).

Multiple logistic regression of tumor penetration on the prognostic factors considered gives significant estimates of odds ratios *γ* for Gleason score ( $\hat{\gamma }$=2.6; *p* < 0.00001), digital rectal exam ( $\hat{\gamma }$=2.1; *p* = 0.0008), and PSA ( $\hat{\gamma }$=1.03; *p* = 0.008).

Despite impressive odds ratios and p‐values, the proportion of variation attributable to the set of seven prognostic factors is only moderate, *EV* = 0.30, accompanied by moderately large and balanced degrees of necessity and sufficiency, *DN*_1_ = 0.56 and *DS*_1_ = 0.52, respectively. Detailed results for the individual prognostic factors are provided by Table 6. For comparison, we also cite *DN*_2_ and *DS*_2_, which only differ for nondichotomous prognostic factors. Personally, we prefer *DN*_1_ and *DS*_1_ because of their closer connection to *EV*. Besides Equation (7), we recall from Section 2 that *EV* = *DN*_1_ · *DS*_1_ for dichotomous prognostic factors. Approximately, this relationship is also observed for the ordinal or continuous prognostic factors age, digital rectal exam, PSA, tumor volume, and Gleason score (all modeled linearly).

**Table 6:** Marginal degrees of necessity and sufficiency and explained variation of prognostic factors for tumor penetration of prostatic capsule

Prognostic factors	EV	DN_1_	DS_1_	DN_2_	DS_2_
Age	<0.01	0.05	0.04	0.04	0.03
Digital rectal exam	0.09	0.49	0.20	0.49	0.12
PSA	0.12	0.23	0.46	0.21	0.34
Tumor volume	0.01	0.16	0.08	0.12	0.07
Gleason score	0.23	0.51	0.44	0.47	0.39
Race	<0.01	0.03	<0.01	0.03	<0.01
Capsular involvement	0.06	0.10	0.58	0.10	0.58
**Full model**	0.30	0.56	0.52	0.50	0.43

In terms of marginal *EV*, the Gleason score is the strongest single prognostic factor, followed by PSA, digital rectal exam, and capsular involvement. Due to the different scales of the prognostic factors, such a ranking would have been difficult to establish from odds ratios. We now go a step further and decompose *EV* values into corresponding degrees of necessity and sufficiency. The strongest prognostic factor, Gleason score, is reasonably strong in both *DN* and *DS* (0.51 and 0.44, respectively). With respect to sufficiency—*if you have the harmful level, it is difficult to avoid the unfavorable outcome*—the detection of capsular involvement with *DS*_1_ = 0.58 attracts attention; however, its degree of necessity is remarkably low (*DN*_1_ = 0.10). The digital rectal exam stands out with respect to its degree of necessity with *DN*_1_ = 0.49—*without a harmful level, the unfavorable outcome becomes not very likely*—but its degree of sufficiency being rather low (*DS*_1_ = 0.20).

Only the BCa confidence interval for the lowest *DN*_*1*_ and *DS*_*1*_ values of age and race includes 0. *DN*_*1*_ and *DS*_*1*_ for the full model are 0.56 (95% confidence interval 0.48 to 0.63) and 0.52 (0.43 to 0.59), respectively.

Regarding partial results, only the Gleason score shows a considerable partial *EV* of 0.09. Interestingly, this variable hardly contributes to the *DS* of the full model (partial *DS*_1_ = 0.03) while it contributes about one third of the *DN* of the full model (partial *DN*_1_ = 0.16).

### 5.2 Prognostic factors for survival with primary biliary cirrhosis

A randomized trial in primary biliary cirrhosis of the liver was conducted at the Mayo Clinic from 1974 to 1984. For a total of 312 patients, survival times (time until death from any cause) and status (60% censored) as well as several prognostic factors have been obtained and analyzed by Fleming and Harrington.^26^ In our analysis, we used the following prognostic factors: age (in years), presence of edema (no, yes), albumin (in mg/dl, range 2.0‐4.6), log of serum bilirubin (serum bilirubin in mg/dl, range 1.2‐3.3), and log of prothrombin time (prothrombin time in seconds, range 2.2‐2.8). The time range of medical interest, adequately covered by the sample, is 0 to 12 years.

Multiple Cox regression gives significant estimates of hazard ratios *γ* for log of bilirubin ( $\hat{\gamma }$=2.4; p < 0.00001), albumin ( $\hat{\gamma }$=0.37; *p* < 0.0001), age ( $\hat{\gamma }$=1.03; *p* = 0.0001), and log of prothrombin time ( $\hat{\gamma }$=25.6; *p* = 0.001).

The proportion of the variation of survival explained by the full model is 0.40, which is relatively high for studies of survival (cf Table 3 in the work of Schemper and Henderson^18^). The *EV* of 0.40 is accompanied by relatively large and balanced degrees of necessity and sufficiency (*DN*_1_ = 0.63 and *DS*_1_ = 0.59).

Hazard ratios and p‐values of the individual prognostic factors are quite impressive. Since they are measured on different scales, their importance should be compared using the *EV* values for the individual prognostic factors in Table 7.

**Table 7:** Marginal degrees of sufficiency and necessity and explained variation of prognostic factors for survival with primary biliary cirrhosis

**Prognostic factors**	**EV**	**DN**_**1**_	**DS**_**1**_	**DN**_**2**_	**DS**_**2**_
log (bilirubin)	0.31	0.56	0.51	0.52	0.43
log (prothrombin time)	0.09	0.28	0.24	0.25	0.17
Edema	0.10	0.20	0.53	0.20	0.53
Albumin	0.17	0.41	0.36	0.35	0.28
Age	0.06	0.29	0.20	0.25	0.16
**Full model**	0.40	0.63	0.59	0.59	0.50

**Figure 2:**
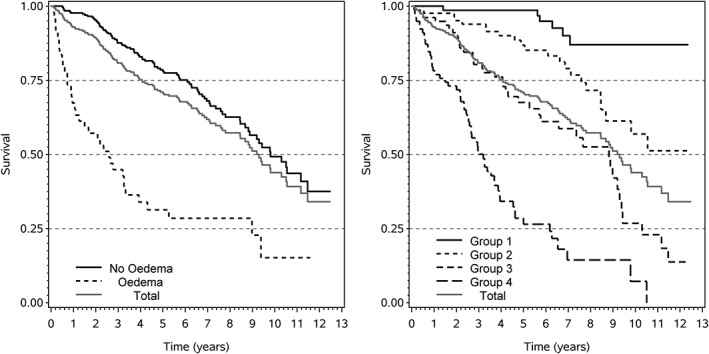
The effect of edema (left panel) and of log bilirubin (right panel) on survival with primary biliary cirrhosis. Log bilirubin grouped according to quartiles

Edema seems to be quite sufficient for death of these patients (*DS*_1_ = 0.53), but the much lower degree of necessity (*DN*_1_ = 0.20) leads to a small proportion of explained variation (*EV* = 0.10). The left panel of Figure 2 shows that the survival outlook of the small subgroup of patients with edema (16% of patients) is much worse. It is relatively hard to escape death, once edema is observed. In contrast, survival probabilities for patients without edema are close to the total average at each time point, resulting in relatively low necessity.

Log bilirubin is by far the strongest prognostic factor and accounts for about three quarters (*EV* = 0.31) of the explanatory capacity of the full model with *EV* = 0.40. The prognostic factor receives similarly strong contributions from necessity (*DN*_1_ = 0.56) and sufficiency (*DS*_1_ = 0.51). This is confirmed by the right panel of Figure 2, which indicates an approximately symmetric distribution of conditional survival probabilities around the unconditional probabilities at most time points.

Log bilirubin also is the only prognostic factor with nonnegligible partial *DN* and *DS* values (partial *DN*_1_ = 0.14, partial *DS*_1_ = 0.09).

## 6 CONCLUDING REMARKS

The suggested measures *DN* and *DS* comply with the desirable properties for such measures postulated in Section 1:

1. They conform to property 1 with respect to simplicity, intuitive appeal, and the well interpretable range of values (see Section 2.1). They share this property with *AR*, which they extend in various ways.
2. With respect to property 2, *DN* and *DS* share the same structure. However, they are independently sensitive to relevant characteristics of a dataset, as demonstrated empirically in Section 4. In Appendix B, we show that all pairs of values (*DN*, *DS*) are possible within [0, 1]^2^.
3. The correspondence between *DN* and *DS* on one side and *EV* on the other (property 3) is confirmed for both variants of *DN* and *DS* at the extremes of their scales (0 and 1; see Section 2.3). Only for the first variants, *DN*_1_ and *DS*_1_, the intuitive relationship (7) has been deduced for the full scale [0, 1]. Furthermore, *EV* = *DN* · *DS* has been proven for the case of a single dichotomous prognostic factor, while for general types of prognostic factors, an approximate equality of *DN*_1_ · *DS*_1_ and *EV* has been demonstrated empirically.
4. Properties 4 and 5 are fulfilled since only unconditional and conditional predictions are required. They may be obtained from models of any type including prognostic factors of any type.

The notions of necessary and sufficient conditions for events to happen have existed for a long time both in formal logic as well as in empirical sciences. Though potentially fruitful for increasing knowledge of cause‐effect relationships in medicine, related concepts have been a neglected field in biostatistics. We anchor our proposed measures of the degrees of necessity and of sufficiency between *attributable risk* and *explained variation*. Both of them are well established concepts in biostatistics. The attributable risk for a dichotomous prognostic factor is at the core of our more general measure of the degree of necessity. We have introduced a new measure of the degree of sufficiency that is as symmetric to the measure of the degree of necessity as the original notions are. Together, both measures have been linked to an established measure of the degree of variation in the outcome, explained by a prognostic factor. Explained variation, already part of the toolbox for the analysis of prognostic factor studies, measures the “importance” of prognostic factors,^27^ which carries independent information, additional to odds or hazard ratios and p‐values from regression. Our suggested measures provide additional information on *EV*‐values, whether these are primarily due to a factor's contribution to necessity, to sufficiency, or to both in a balanced way.

Because of this feature, our measures have been designed to share relevant properties with a measure of explained variation. In particular, while suitable for use in connection with a modeling task by logistic and Cox regression, they do not require such a model. They are only based on unconditional and conditional probability estimates of the outcomes, and these might come from other regression models (eg, probit regression or parametric Weibull survival models), from neural networks, expert judgment, or clinical classification schemes. In any case, the application of our proposed measures is conditional on the appropriateness of the specified relationship between prognostic factors and outcome.

The degrees of necessity and sufficiency can be compared across different types of models, different types of prognostic factors (categorical or continuous), and prognostic indices with a different number of prognostic factors. By means of partial *DN* and *DS*, the benefit of adding a new prognostic factor to an already existing group of established factors can be judged ‐ similar to a prognostic factor's additional contribution to *EV*. If, in a medical application, either *only the increase in necessity* or *only the increase in sufficiency* is of interest, the selection of prognostic factors could be controlled by the gain in *DN* or in *DS*, rather than by the usual more general criteria.

Just as with *EV*, also the estimation of *DN* and *DS* relies on data from representative samples with respect to the distributions of the prognostic factors and, even more, of the outcome variable. The outcome distribution might be reliably obtained from cross‐sectional or cohort, but not from case‐control studies.

Though the simple measurement scale of the four measures presented makes them equally easy in interpretation, we prefer *DN*_1_ and *DS*_1_ because of their closer relationship with explained variation.

Application of the suggested measures is facilitated by an R function *NecSuff* and by a SAS macro *NecSuff*, both of which are available in the online supplement.

## Supporting information

SIM_8331‐Supp‐0001‐Gleiss_Schemper_Supplement_revised.pdf

## APPENDIX A

To show that *EV* ≥ *DN*_2_ · *DS*_2_, we use definitions (3), (4), and (6)

Due to the convexity of the squaring function (Jensen's inequality), this results in

(A1) $$\frac{EV}{{DN}_{2}\cdot {DS}_{2}}\geq \left(1-\alpha \right)\frac{E_{X<}\left(P\left(D\right)-P(D\;|\;X)\right)}{E_{X>}\left(P\left(D\;|\;X\right)-P\left(D\right)\right)}+\alpha \frac{E_{X>}\left(P\left(D\;|\;X\right)-P\left(D\right)\right)}{E_{X<}\left(P\left(D\right)-P(D\;|\;X)\right)}.$$

Since E_*X*_(P(*D* | *X*)) = P(*D*) and E_*X*_(P(*D* | *X*)) = (1 − *α*)E_*X*<_(P(*D* | *X*))+*α*E_*X*>_(P(*D* | *X*)), we get (1 − *α*)*E*_*X*<_(P(*D*) − P(*D* | *X*)) − *αE*_*X*>_(P(*D* | *X*) − P(*D*)) = 0. This results in the right‐hand side of (A1) being 1, which proves that *EV* ≥ *DN*_2_ · *DS*_2_.

## APPENDIX B

In order to show that all pairs of values (*DN*, *DS*) within [0, 1]^2^ are possible, let us consider a given pair of values (*DN^∗^*, *DS^∗^*). Let P(*D* | *X*) = *r* for all P(*D* | *X*) < P(*D*) and P(*D* | *X*) = *s* for all P(*D* | *X*) > P(*D*), with *s* > *r* and P(*D*) = (1 − *α*)*r*+*αs*. Inserting into (8) and (9) gives *DN* = *α*(*s* − *r*)/((1 − *α*)*r*+*αs*) and *DS* = (1 − *α*)(*s* − *r*)/(1 − (1 − *α*)*r* − *αs*). The equations *DN* = *DN*^∗^ and *DS* = *DS*^∗^ have solutions

$$r=\frac{\alpha {DS}^{*}\left(1-{DN}^{*}\right)}{\left(1-\alpha \right){DN}^{*}+\alpha {DS}^{*}}$$

and

$$s=\frac{{DS}^{*}\left(\left(1-\alpha \right){DN}^{*}+\alpha \right)}{\left(1-\alpha \right){DN}^{*}+\alpha {DS}^{*}}.$$

These solutions are within [0,1] except if *DN^∗^ = DS^∗^ =* 0, which case corresponds to arbitrary *r* = *s*.
