# Supplementary material for: Quantifying degrees of necessity and of sufficiency in cause‐effect relationships with dichotomous and survival outcomes
Source: Stat Med. 2019 Aug 6;38(23):4733–48. doi: 10.1002/sim.8331 (PMC6771968; doi:10.1002/sim.8331)
Supplement: Supplementary file 1 — SIM_8331‐Supp‐0001‐Gleiss_Schemper_Supplement_revised.pdf [file SIM-38-4733-s001.pdf]

Quantifying degrees of necessity and of sufficiency in cause-effect relationships with dichotomous and survival outcomes - Supplementary material

Andreas Gleiss, Michael Schemper

Section for Clinical Biometrics, Center for Medical Statistics, Informatics, and Intelligent Systems, Medical University of Vienna, Austria

SUPPLEMENTARY TABLE 1: Small sample bias for estimating *DN* and *DS* for dichotomous outcome and dichotomous prognostic factor, summarized by median (quartiles), for selected scenarios in Table 3.

				Population		***Bias (n=200)***		***Bias (n=500)***	
**Scen.**	**P(*D*)**	***α***	***OR***	***DN***	***DS***	***DN***	***DS***	***DN***	***DS***
1	0.1	0.1	10	0.346	0.346	0.00 (-0.07; 0.07)	-0.01 (-0.08; 0.09)	0.00 (-0.04; 0.04)	0.00 (-0.05; 0.05)
2	0.1	0.5	10	0.787	0.087	0.01 (-0.10; 0.10)	0.00 (-0.02; 0.01)	0.00 (-0.06; 0.06)	0.00 (-0.01; 0.01)
3	0.1	0.9	10	0.878	0.011	0.12 (0.12; 0.12)	0.00 (0.00; 0.00)	0.12 (-0.08; 0.12)	0.00 (0.00; 0.00)
4	0.5	0.1	10	0.087	0.787	0.00 (-0.01; 0.01)	0.01 (-0.08; 0.11)	0.00 (-0.01; 0.01)	0.01 (-0.06; 0.06)
5	0.5	0.5	10	0.519	0.519	0.00 (-0.04; 0.05)	0.00 (-0.04; 0.05)	0.00 (-0.03; 0.03)	0.00 (-0.03; 0.03)
6	0.5	0.9	10	0.787	0.087	0.01 (-0.08; 0.11)	0.00 (-0.01; 0.01)	0.01 (-0.05; 0.05)	0.00 (-0.01; 0.01)
7	0.9	0.1	10	0.011	0.878	0.00 (0.00; 0.00)	0.12 (0.12; 0.12)	0.00 (0.00; 0.00)	0.12 (-0.08; 0.12)
8	0.9	0.5	10	0.087	0.787	0.00 (-0.02; 0.01)	0.00 (-0.09; 0.10)	0.00 (-0.01; 0.01)	0.00 (-0.06; 0.06)
9	0.9	0.9	10	0.346	0.346	0.01 (-0.06; 0.09)	0.01 (-0.07; 0.10)	0.00 (-0.05; 0.05)	0.01 (-0.03; 0.05)
28	0.1	0.1	1	0.000	0.000	0.14 (0.05; 0.47)	0.01 (0.01; 0.05)	0.09 (0.03; 0.29)	0.01 (0.00; 0.02)
29	0.5	0.5	1	0.000	0.000	0.05 (0.02; 0.08)	0.05 (0.02; 0.08)	0.03 (0.01; 0.05)	0.03 (0.01; 0.05)

Abbreviations: P(*D*), unconditional disease probability; *OR*, odds ratio; α, probability of harmful level of *X*. Results based on 500 simulated samples and presented as medians (quartiles).

SUPPLEMENTARY TABLE 2: Small sample bias for estimating *DN* and *DS* for dichotomous outcome and continuous prognostic factor, summarized by medians (quartiles), for the scenarios in Fig. 1, and additionally for *OR*=1.

		Population		***Bias (n=200)***		***Bias (n=500)***	
**P(*D*)**	***OR***	DN_1	DS_1	DN_1	DS_1	DN_1	DS_1
0.5	1	0.00	0.00	0.05 (0.02; 0.08)	0.05 (0.02; 0.08)	0.03 (0.01; 0.05)	0.03 (0.01; 0.05)
0.5	10	0.67	0.67	0.00 (-0.03; 0.03)	0.01 (-0.02; 0.04)	0.00 (-0.02; 0.02)	0.00 (-0.02; 0.02)
0.5	100	0.82	0.82	0.00 (-0.02; 0.03)	0.01 (-0.02; 0.03)	0.00 (-0.01; 0.02)	0.00 (-0.01; 0.01)
0.9	1	0.00	0.00	0.02 (0.01; 0.03)	0.15 (0.07; 0.23)	0.01 (0.00; 0.02)	0.08 (0.04; 0.14)
0.9	10	0.36	0.82	0.01 (-0.06; 0.06)	0.01 (-0.03; 0.04)	0.00 (-0.04; 0.03)	0.00 (-0.02; 0.02)
0.9	100	0.56	0.93	0.00 (-0.06; 0.06)	0.00 (-0.02; 0.02)	0.00 (-0.04; 0.04)	0.00 (-0.01; 0.01)

Abbreviations: P(*D*), unconditional disease probability; *OR*, odds ratio. Results based on 250 simulated samples and presented as median (quartiles).

SUPPLEMENTARY TABLE 3: Small sample bias for estimating *DN* and *DS* for censored survival outcome and balanced dichotomous prognostic factor

		Type 1 censoring								Administrative censoring				
			Population		Bias (n=200)		Bias (n=500)				Bias (n=200)		Bias (n=500)	
HR	τ	% cens.	*DN*	*DS*	*DN*	*DS*	*DN*	*DS*		% cens.	*DN*	*DS*	*DN*	*DS*
1	-	0	0.00	0.00	0.03 (0.04)	0.04 (0.06)	0.02 (0.03)	0.03 (0.04)						
	1.64	20	0.00	0.00	0.04 (0.04)	0.03 (0.04)	0.03 (0.03)	0.02 (0.03)		50	0.05 (0.06)	0.04 (0.04)	0.03 (0.08)	0.03 (0.06)
	0.22	80	0.00	0.00	0.10 (0.14)	0.01 (0.02)	0.07 (0.07)	0.01 (0.01)		90	0.15 (0. 32)	0.02 (0.04)	0.09 (0.19)	0.01 (0.02)
2	-	0	0.22	0.31	0.00 (0.05)	0.00 (0.06)	0.00 (0.04)	0.00 (0.05)						
	2.26	23	0.25	0.20	0.01 (0.08)	0.01 (0.06)	0.00 (0.05)	0.00 (0.04)		50	0.00 (0.08)	0.00 (0.07)	0.00 (0.06)	0.00 (0.06)
	0.30	80	0.32	0.04	0.02 (0.22)	0.00 (0.02)	-0.01 (0.12)	0.00 (0.02)		90	0.00 (0.28)	0.00 (0.03)	-0.01 (0.15)	0.00 (0.02)
10	-	0	0.55	0.62	0.00 (0.04)	0.00 (0.04)	0.00 (0.03)	0.00 (0.03)						
	4.90	31	0.71	0.44	0.00 (0.06)	0.00 (0.04)	0.00 (0.04)	0.00 (0.02)		50	0.00 (0.09)	0.00 (0.06)	0.00 (0.05)	0.00 (0.04)
	0.43	81	0.80	0.09	0.00 (0.11)	0.00 (0.02)	0.00 (0.07)	0.00 (0.02)		90	0.01 (0.16)	0.00 (0.05)	0.01 (0.10)	0.00 (0.02)
100	-	0	0.67	0.69	-0.01 (0.04)	0.00 (0.04)	0.00 (0.02)	0.00 (0.02)						
	15.30	43	0.96	0.45	0.00 (0.03)	0.00 (0.03)	0.00 (0.02)	0.00 (0.02)		50	0.00 (0.03)	0.01 (0.04)	0.00 (0.02)	0.00 (0.03)
	0.47	81	0.98	0.11	0.02 (0.04)	0.00 (0.02)	0.00 (0.03)	0.00 (0.02)		90	0.02 (0.04)	0.00 (0.05)	0.02 (0.04)	0.00 (0.03)

Abbreviations: HR, hazard ratio; τ, time of maximum follow-up; % cens., percentage of censored observations. Results based on 250 simulated samples and presented as medians (inter-quartile ranges).

SUPPLEMENTARY TABLE 4: Small sample bias for estimating *DN* and *DS* for uncensored survival outcome and dichotomous prognostic factor for the scenarios of Table 5.

		Population		Bias (n=200)		Bias (n=500)	
	HR	*DN*	*DS*	*DN*	*DS*	*DN*	*DS*
α = 0.1	1	0.00	0.00	0.02<br>(0.01; 0.08)	0.04<br>(0.02; 0.18)	0.01<br>(0.01; 0.05)	0.02<br>(0.01; 0.09)
	2	0.05	0.47	0.00<br>(-0.01; 0.01)	0.01<br>(-0.07; 0.06)	0.00<br>(-0.01; 0.01)	-0.01<br>(-0.05; 0.03)
	10	0.16	0.85	0.00<br>(-0.02; 0.02)	0.00<br>(-0.03; 0.02)	0.00<br>(-0.01; 0.01)	0.00<br>(-0.01; 0.01)
	100	0.24	0.94	0.01<br>(-0.02; 0.04)	-0.01<br>(-0.02; 0.00)	0.00<br>(-0.02; 0.03)	0.00<br>(-0.01; 0.00)
α = 0.5	1	0.00	0.00	0.03<br>(0.01; 0.05)	0.04<br>(0.02; 0.08)	0.02<br>(0.01; 0.04)	0.03<br>(0.01; 0.05)
	2	0.22	0.31	0.00<br>(-0.02; 0.03)	0.00<br>(-0.03; 0.03)	0.00<br>(-0.02; 0.02)	0.00<br>(-0.03; 0.02)
	10	0.55	0.61	0.00<br>(-0.02; 0.02)	0.00<br>(-0.02; 0.02)	0.00<br>(-0.02; 0.01)	0.00<br>(-0.02; 0.01)
	100	0.68	0.68	-0.01<br>(-0.03; 0.01)	0.00<br>(-0.02; 0.02)	0.00<br>(-0.01; 0.01)	0.00<br>(-0.01; 0.01)
α = 0.9	1	0.00	0.00	0.02<br>(0.01; 0.08)	0.04<br>(0.01; 0.13)	0.02<br>(0.01; 0.06)	0.02<br>(0.01; 0.08)
	2	0.36	0.08	-0.01<br>(-0.09; 0.07)	0.00<br>(-0.03; 0.02)	-0.01<br>(-0.06; 0.05)	0.00<br>(-0.02; 0.01)
	10	0.80	0.21	0.00<br>(-0.04; 0.03)	-0.01<br>(-0.03; 0.02)	0.00<br>(-0.03; 0.02)	0.00<br>(-0.02; 0.01)
	100	0.93	0.25	-0.01<br>(-0.03; 0.00)	0.00<br>(-0.03; 0.02)	0.00<br>(-0.01; 0.01)	0.00<br>(-0.02; 0.01)

Abbreviations: HR, hazard ratio; α, probability of harmful level of *X*. Results based on 250 simulated samples and presented as medians (quartiles).

Programming Code

In the following we provide a SAS macro and an R function to calculate DN and DS for dichotomous outcomes. See http://cemsiis.meduniwien.ac.at/en/kb/science-research/software/statistical-software for latest versions as well as for additional functions for survival.

SAS Macro

```
*******************************************************************;
*
* NECSUFF
* =======
*
* SAS-macro for computation of degrees of necessity and sufficiency
*
* Gleiss, A. & Schemper, M., Quantifying degrees of necessity and
* sufficiency in cause-effect relationships with dichotomous and
* survival outcome
* submitted to Stat.Med.
*
* Author:  Andreas Gleiss
* Version: 1.0
* Date:    8 August 2018
*
* Macro parameters:
* =================
*
* data      name of SAS data set
* y         name of dichotomous outcome variable
* x         name of independent variable (or linear predictor)
* refcat    reference category of y (first=default or last)
* inpred    dataset containing predictions
* inpredvar name of variable in inpred dataset containing
*           predictions
* odssel    control output from proc logistic
*           (default: ResponseProfile)
* print     =1 to print results (default), =0 (e.g., for simulations)
*
*******************************************************************;

%macro necsuff(data, y, x, refcat=first, inpred=, inpredvar=,
                        odssel=ResponseProfile, print=1);
data _work;
      set &data;
      if missing(&x) or missing(&y) then delete;
      run;
%if &inpred= %then %do;
      ods select &odssel;
      proc logistic data=_work outest=_betas;
            class &y / param=ref ref=&refcat;
            model &y=&x;
            output out=_est1 pred=p_i_dach;
            run;
      data _betas;
            set _betas(rename=(intercept=beta0 &x=beta1));
            _dummy=1;
            keep _dummy beta0 beta1;
```

```
            run;
      %end;
%else %do;
      data _est1;
            set &inpred;
            p_i_dach=&inpredvar;
            run;
      data _betas;
            _dummy=1; beta0=.; beta1=.; output;
            run;
      %end;
proc sort data=_est1;
      by descending p_i_dach;
      run;
data _est1;
      set _est1;
      _dummy=1;
      _row=_n_;
      run;
proc means data=_work noprint;
      var &y;
      output out=_my1 mean=p_bar max=_dummy;
      run;
data _est1_;
      merge _est1
              _my1(keep=p_bar _dummy _freq_ rename=(_freq_=n))
              _betas;
      by _dummy;
      retain dn1_sum ds1_sum dn2_sum ds2_sum n_smaller n_larger (0 0 0 0 0 0);

      smaller=(p_i_dach<p_bar);
      larger=(p_i_dach>p_bar);
      n_smaller=n_smaller+smaller;
      n_larger=n_larger+larger;

      if p_bar~=0 then do;
            if smaller then dn1_sum=dn1_sum + ((p_bar - p_i_dach)/p_bar)**2;
            if smaller then dn2_sum=dn2_sum + ((p_bar - p_i_dach)/p_bar);
            end;
      if p_bar~=1 then do;
            if larger then ds1_sum=ds1_sum + ((p_i_dach - p_bar)/(1-
p_bar))**2;
            if larger then ds2_sum=ds2_sum + ((p_i_dach - p_bar)/(1-p_bar));
            end;
      run;

data _necsuff_;
      set _est1_;
      where _row=n;
      if n_smaller~=0 then do;
            dn_1_=dn1_sum/n_smaller;
            dn_2=dn2_sum/n_smaller;
            end;
      if n_larger~=0 then do;
            ds_1_=ds1_sum/n_larger;
            ds_2=ds2_sum/n_larger;
            end;
      weight_dn=p_bar/(1-p_bar) * n_smaller/n;
```

```
      weight_ds=(1-p_bar)/p_bar * n_larger/n;
      ev_indir=weight_dn*dn_1_ + weight_ds*ds_1_;
      dn_1=sqrt(dn_1_);
      ds_1=sqrt(ds_1_);
      alpha=n_larger/n;
      or=exp(beta1);
      progn_fact="&x";
      keep progn_fact p_bar alpha or ev_indir dn_1 ds_1 dn_2 ds_2;
      run;
ods select all;
%if &print=1 %then %do;
      title "Degrees of necessity and sufficiency";
      title2 "Outcome = &y";
      proc print data=_necsuff_ noobs label;
            var progn_fact p_bar alpha or ev_indir dn_1 ds_1 dn_2 ds_2;
            format or p_bar alpha ev_indir dn: ds: f5.3;
            label       progn_fact="Prognostic factor"
                        or="OR" ev_indir="EV" p_bar="est.P(D)"
                        dn_1="DN1" ds_1="DS1" dn_2="DN2" ds_2="DS2";
            run;
      title; title2;
      %end;
%mend;

*%necsuff(data=sh_tab3.hl_prost, y=capsule, x=psa);
*%necsuff(data=sh_tab3.hl_prost, y=capsule, x=gleason, odssel=none);

data lungca; * Swedish cohort study (Nilsson et al, 2001);
      do i=1 to 8120;
            x=0; y=0; output;
            end;
      do i=1 to 36;
            x=0; y=1; output;
            end;
      do i=1 to 4331;
            x=1; y=0; output;
            end;
      do i=1 to 177;
            x=1; y=1; output;
            end;
      run;
%necsuff(data=lungca, y=y, x=x);
```

R function

```
#####################################################################
#
# NecSuff
# =======
#
# R-function for computing degrees of necessity and sufficiency
#
# Gleiss, A. & Schemper, M. Quantifying degrees of necessity and
# sufficiency in cause-effect relationships with dichotomous and
# survival outcome,
# submitted to Stat.Med.
#
# Author:  Andreas Gleiss
# Version: 1.0
# Date:    09 Aug 2018
#
# Arguments:
# ==========
#
# pred     name of variable containing predictions
#
#####################################################################

NecSuff<-function(pred){
  p_bar<-mean(pred)
  smaller<-(pred<p_bar)
  larger<-(pred>p_bar)
  DN1<-sqrt(mean(((p_bar-pred[smaller])/p_bar)^2))
  DS1<-sqrt(mean(((pred[larger]-p_bar)/(1-p_bar))^2))
  DN2<-mean((p_bar-pred[smaller])/p_bar)
  DS2<-mean((pred[larger]-p_bar)/(1-p_bar))

  w_dn<-p_bar/(1-p_bar) * sum(smaller)/length(pred)
  w_ds<-(1-p_bar)/p_bar * sum(larger)/length(pred)
  EV<-w_dn*DN1^2 + w_ds*DS1^2

  cat('\nest.P(D) =',round(p_bar,3))
  cat('\nDN1 =',round(DN1,3),', DS1 =',round(DS1,3))
  cat('\nDN2 =',round(DN2,3),', DS1 =',round(DS2,3))
  cat('\nEV =',round(EV,3))
}

xx<-rnorm(1000)
pp<-1/(1+exp(-5-log(5)*xx))
yy<-1*(runif(1000)<=pp)
plot(yy~xx)
points(cbind(xx,pp),col="red")
res.glm<-glm(yy~xx, family=binomial)
```

```
summary(res.glm)
pred<-predict(res.glm, type="response")
NecSuff(pred)

# Swedish cohort (Nilsson et al, 2001)
xx<-c(rep(0,36),rep(1,177),rep(0,8120),rep(1,4331))
yy<-c(rep(1,36),rep(1,177),rep(0,8120),rep(0,4331))
table(yy,xx)
res.glm<-glm(yy~xx, family=binomial)
summary(res.glm)
pred<-predict(res.glm, type="response")
NecSuff(pred)
```
